# International Medical Annual and Practitioners’ Index

**Published:** 1892-06

**Authors:** 


The International Medical Annual and Practitioners' Index: A Work of Reference for Medical Practitioners. Editor, Percy Wilde, M. D.; Editorial Secretary, P. Watson Williams, M. D. 1892. Tenth year. New York: E. B. Treat, 5 Cooper Union. Chicago: 199 Clark Street. Price, $2.75.

This well-bound volume of six hundred and fifty pages, printed on excellent paper, giving the progress of the medical sciences for 1891, is out. The editors and contributors to this issue are men of experience from different parts of the world, and have done their work well. That the Annual has reached its tenth year and has been increasing in size from year to year, is in itself a recommendation. The cuts and plates that are scattered throughout the book are of the best, and the whole volume makes the impression of stability.

Part I Dr. Wilde starts out with a "Dictionary of the New Remedies," and a "Review of Therapeutics for 1891." He gives an extended review of the uses of the new remedies and new indications for old remedies. Part II contains a "Dictionary of New Treatment in Medicine and Surgery. 1892." In alphabetical order, beginning with abdominal injuries and ending with yellow fever, we get in a nut-shell, as it were, the recent advances in the treatment of all the ailments humanity is subject to—medical, surgical, gynæcological, and dermatological. Part III gives the "Recent Advances in Bacteriology." There is a paragraph devoted to "Medical Photography," one to "Sanitary Science," and one to "The Use of Suppositories in the Treatment of Disease." A few pages are devoted to "Improvements in Pharmacy," and lastly, "New Inventions, Instruments, and Appliances." There is a list of medical books published during 1891. A general index concludes the work. This index is very complete and enables one to find at a glance what we are looking for. W. S.

